# The Closure of Grade 1 Coronary Perforation by the Snowplow Phenomenon

**DOI:** 10.7759/cureus.54818

**Published:** 2024-02-24

**Authors:** Dibyasundar Mahanta, Anindya Banerjee, Abhinav Kumar, Pranjit Deb, Kowtarapu Sai Karthik, Saran P Mohanan, Sindhu Rao Malla, Debasish Das

**Affiliations:** 1 Cardiology, SUM Hospital, Bhubaneswar, IND; 2 Cardiology, All India Institute of Medical Sciences, Bhubaneswar, Bhubaneswar, IND; 3 Cardiology, All India Institute of Medical Sciences, Bhubaneswar, IND

**Keywords:** phenomenon, snow plow, perforation, coronary, closure

## Abstract

We report an extremely rare case of spontaneous closure of grade 1 coronary perforation by the snowplow phenomenon during the revascularization of a subtotal occlusion in the left anterior descending coronary artery. Coronary artery perforation is usually a nightmare during coronary intervention in the cardiac catheterization laboratory. While large coronary perforation requires the deployment of a covered stent, small perforations require heparin reversal, prolonged balloon inflation, deployment of small coils, or gel foam closure. The coronary segment with a small perforation was stented with a drug-eluting stent (DES), which might have resulted in the shifting of the fatty plaque toward the perforation and subsequently sealing the coronary perforation.

## Introduction

Coronary perforation is a nightmare in the cardiac catheterization laboratory during coronary intervention. Large coronary perforation results in rapid cardiac tamponade with hemodynamic collapse and carries a risk of sudden cardiac arrest. Early recognition and management of coronary perforation is the key to preventing hemodynamic compromise and cardiac arrest in the patient. Large coronary perforations are managed with the deployment of covered stents across the perforation segments [1]. Small coronary perforations can result in delayed development of cardiac tamponade after four to six hours. Small-vessel coronary perforations require heparin reversal with protamine, prolonged balloon inflation, and deployment of small coils or gel foam embolization. We report a rare case in which a small coronary perforation was sealed off by the displaced fatty plaque with the deployment of a drug-eluting stent.

## Case presentation

**Figure 1:**
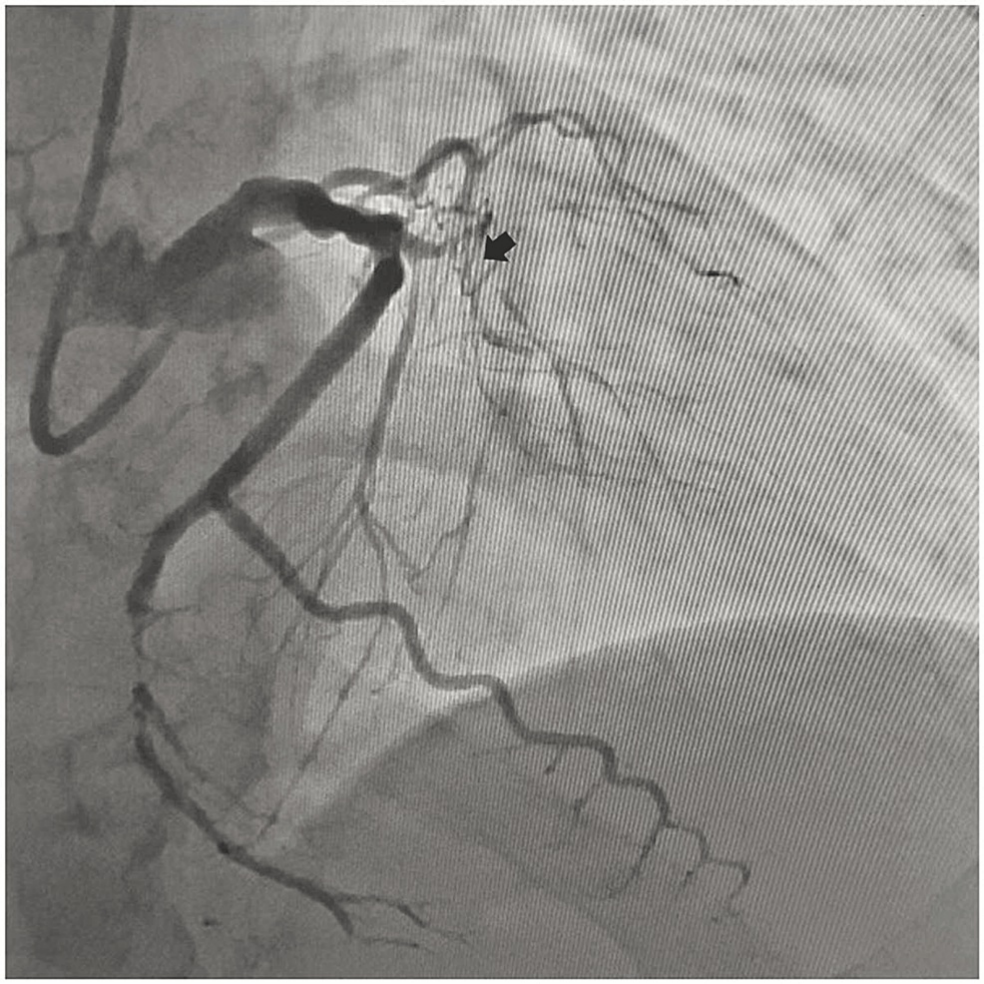
Calcific subtotal occlusion in the proximal left anterior descending coronary artery (LAD).

**Figure 2:**
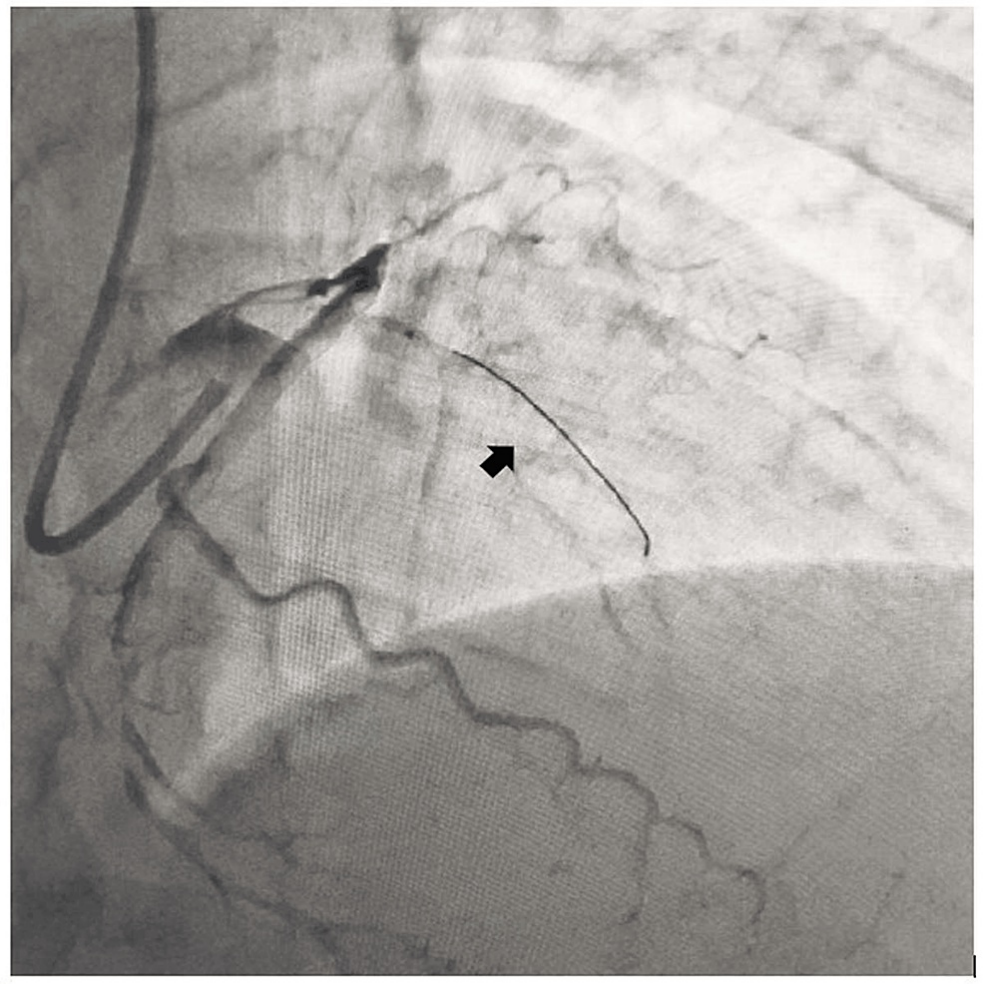
Antegrade crossing of subtotal occlusion in left anterior descending coronary artery (LAD) with a 0.014'' Fielder FC wire.

**Figure 3:**
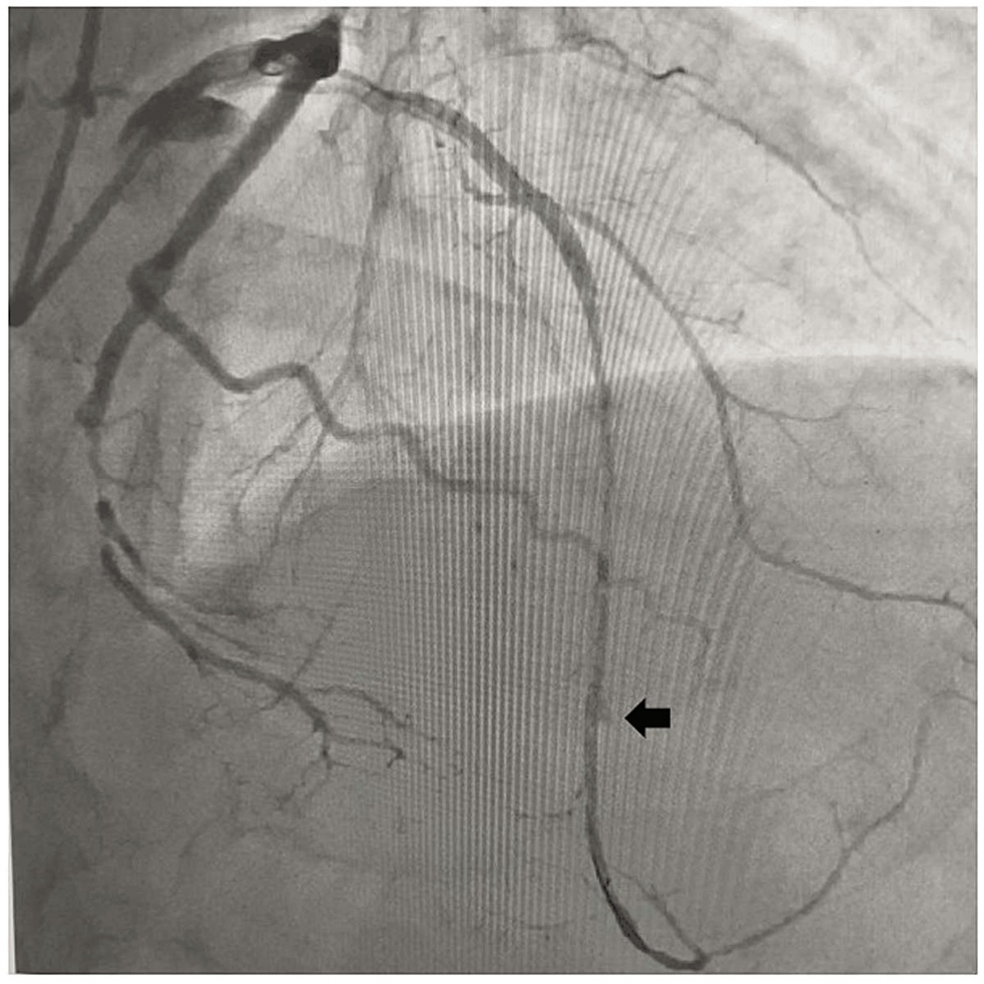
Small (grade 1) coronary perforation in the distal left anterior descending coronary artery (LAD).

**Figure 4:**
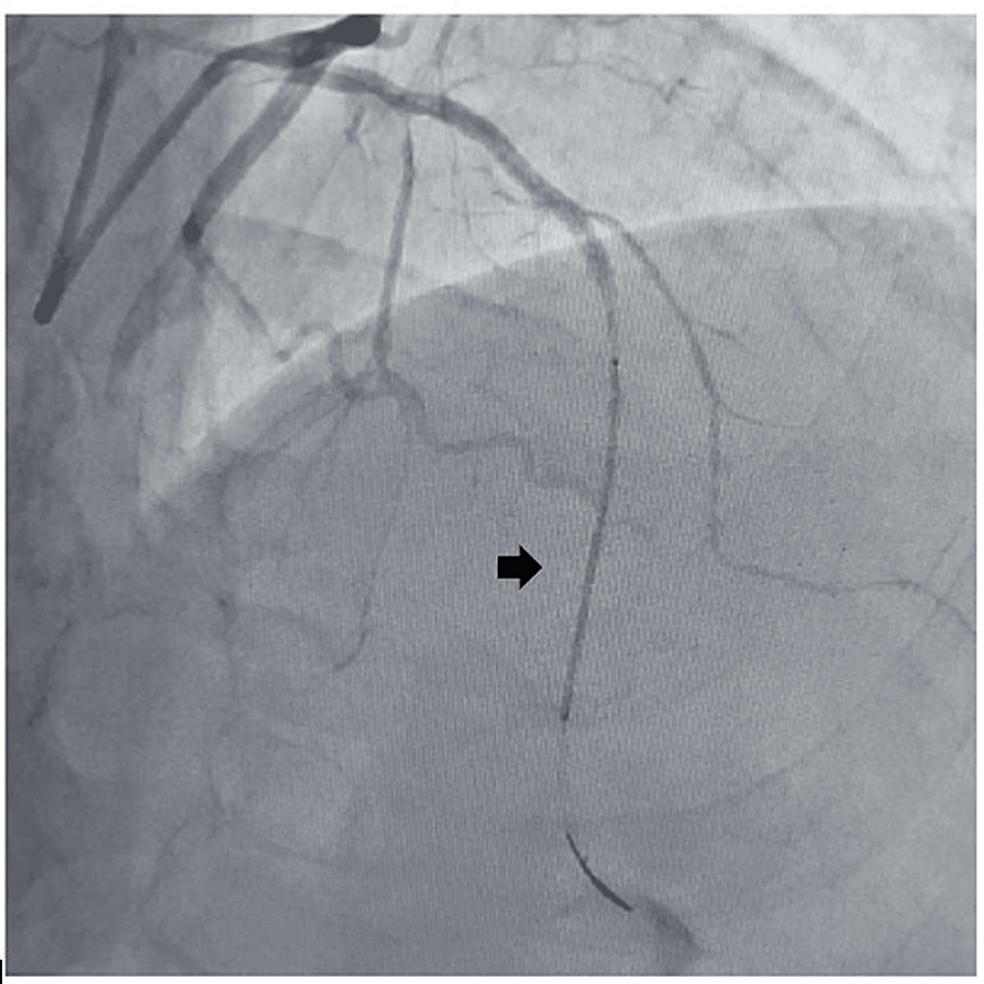
Drug-eluting stent across the perforation segment.

**Figure 5:**
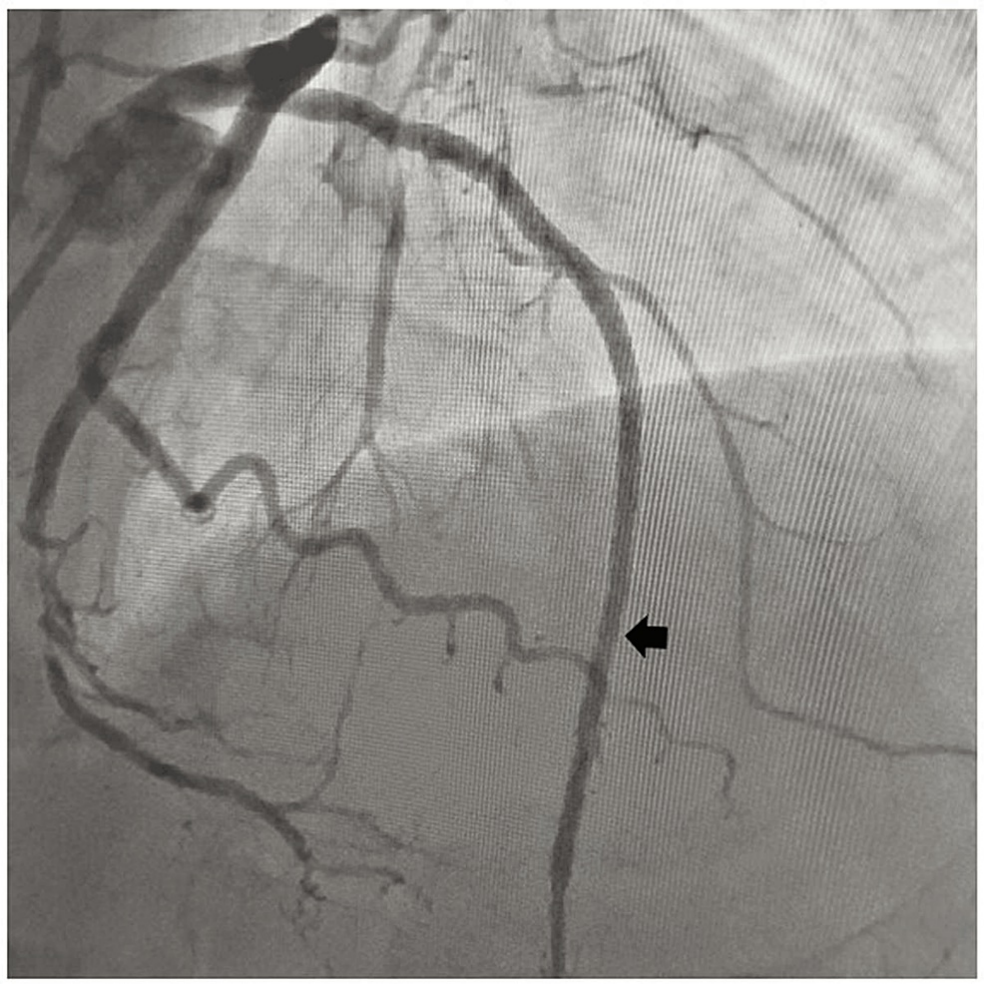
Sealed coronary perforation with snowplow phenomenon.

A 65-year-old male, diabetic, non-hypertensive, and non-smoker, presented to the cardiology outpatient department with rest angina classified as Canadian Cardiovascular Society (CCS) Class IV for the last three days, along with symptoms of shortness of breath and diaphoresis. For the past six months, he had been experiencing effort angina classified as Canadian Cardiovascular Society (CCS) Class II. He had no history of palpitation, presyncope, or syncope. During the examination, his heart rate was 84 beats per minute, and his blood pressure measured 136/80 mmHg in the right arm while in a supine position. Cardiovascular system examination revealed the presence of left ventricular fourth heart sound (S4). The electrocardiogram (ECG) revealed the presence of QS in anteroseptal precordial leads, suggesting an old myocardial infarction. His Troponin-T level was not elevated. Echocardiography revealed the presence of a regional wall motion abnormality in the anterior wall, accompanied by severely depressed left ventricular systolic function (EF = 35%). His blood parameters were within normal limits. Because of ongoing angina for the last three days and the regional wall motion abnormality, he was subjected to a right trans-radial coronary angiogram, which revealed subtotal occlusion of the mid-left anterior descending coronary artery (LAD), with 60% to 70% lesion in the distal circumflex coronary artery (LCX) (Figure 1). The left main coronary artery was accessed using an additional backup guide catheter (EBU) 6F 3.5, and the subtotal occlusion in the LAD was successfully traversed with a 0.014 Fielder FC guide wire assisted by proximal balloon support (Figure 2). The lesion in proximal LAD was predilated with a 2 mm x 10 mm semi-compliant balloon, and the lesion was stented with a 2.75 mm x 32 mm drug-eluting stent (DES) with a good angiographic result. Post-stenting, the distal long lesion in LAD was well visible with perforation in the distal end. It was a grade 1 coronary perforation with local staining of the myocardium (Figure 3). The patient was hemodynamically stable, and there was minimal pericardial effusion in echocardiography. Heparin was reversed with protamine. As a long-covered stent was not available, it was decided to cover the perforated segment with a 2.5 mm x 34 mm DES at 14 atm pressure and post-dilated with a 2.5 mm x 10 mm non-compliant balloon at 18 atm pressure (Figure 4). Following the deployment of the long distal stent, interestingly, it was found that the distal perforation was itself sealed off with the fatty plaque migration (snowplow effect) (Figure 5). Then the mid-LAD was stented with 2.75 mm x 16 mm DES at 16 atm pressure. After stent deployment, the LAD revealed favorable angiographic results, demonstrating distal thrombolysis in myocardial infarction (TIMI) III flow (Figure 5). Our case is an extremely rare illustration of the sealing of small coronary perforation by displacement of the fatty atherosclerotic plaque toward the lumen of the coronary perforation after the deployment of the DES (snowplow phenomenon).

## Discussion

Coronary perforations are a nightmare in the cath lab. Large coronary perforations result in immediate cardiac tamponade with hemodynamic collapse, whereas small coronary perforations result in delayed onset cardiac tamponade. The most common cause of coronary perforation is guide wire-induced perforations, especially with the use of more slippery and hydrophilic guide wires. Small vessels, distal vessels, the presence of tortuosity, dense calcium, inadvertent forward push on the guide wires, and rapid hardwire exchanges are predictors of coronary perforation. In the present case, guide wire negotiation after crossing the subtotal occlusion was blind as the distal vessel was not at all visible with complete occlusion of the lumen with guide wire, resulting in a small coronary perforation. As the coronary perforation was very small (grade 1), the patient was hemodynamically stable without much pericardial effusion. Large coronary perforations are usually managed with covered stents, whereas small coronary perforations are managed with heparin reversal with protamine, prolonged balloon inflation, coiling [2], gel foam closure [3,4], or glue [5]. As a large covered stent was not available, we planned to deploy a conventional DES to cover the distal lesion followed by prolonged balloon inflation if the bleeding continues post-heparin reversal. We stented the perforation segment with a long drug-eluting stent at 16-18 atm pressure after which the bleeding from the side of the LAD itself sealed off due to the snowplow phenomenon (side-wise slipping of the atherosclerotic plaque toward the perforation after coronary stenting). We did not push further heparin in the patient although the procedure was more than one hour as it would disassemble the closed perforation again. We followed up the patient for the next six hours in the intensive care unit, he was hemodynamically stable without any development of pericardial effusion. He was put on dual antiplatelets, statin, beta-blocker, and diuretics postprocedure, and he was doing well during follow-up after one month in the outpatient department. Our case is an interesting and rare case of spontaneous closure of small coronary perforation by snowplow phenomenon after revascularization with a conventional DES. While prolonged balloon inflation is the most common strategy for managing small perforations, conventional stenting can rarely achieve perforation closure through the snowplow phenomenon.

## Conclusions

We report an interesting and rare case of spontaneous closure of a small coronary perforation after conventional stenting with DES, attributed to the snowplow phenomenon. However, we recommend that the two primary strategies to seal coronary perforation are prolonged balloon inflation and the deployment of a covered coronary stent. Although several strategies, such as heparin reversal with protamine, closure with micro-coils, and gel foam embolization, can seal off coronary perforation, the snowplow phenomenon after stenting with a conventional DES can also rarely achieve the same result.
